# Identification of HNF-4α as a key transcription factor to promote ChREBP expression in response to glucose

**DOI:** 10.1038/srep23944

**Published:** 2016-03-31

**Authors:** Jian Meng, Ming Feng, Weibing Dong, Yemin Zhu, Yakui Li, Ping Zhang, Lifang Wu, Minle Li, Ying Lu, Hanbei Chen, Xing Liu, Yan Lu, Haipeng Sun, Xuemei Tong

**Affiliations:** 1 Department of Biochemistry and Molecular Cell Biology, Shanghai Key Laboratory for Tumor Microenvironment and Inflammation, Shanghai Jiao Tong University School of Medicine, 280 S. Chongqing Road, Shanghai, 200025, China; 2 Department of Pathophysiology, Key Laboratory of Cell Differentiation and Apoptosis of Chinese Ministry of Education, Shanghai Jiao Tong University School of Medicine, 280 S. Chongqing Road, Shanghai, 200025, China; 3 Department of Endocrinology, Xinhua Hospital, Shanghai Jiao Tong University School of Medicine, 1665, Kong Jiang Road, Shanghai, 200092, China; 4 Department of Endocrinology and Metabolism, Ruijin Hospital, Shanghai Jiao Tong University School of Medicine, 197, Rui-Jin 2nd Road, Shanghai, 200025, China

## Abstract

Transcription factor carbohydrate responsive element binding protein (ChREBP) promotes glycolysis and lipogenesis in metabolic tissues and cancer cells. ChREBP-α and ChREBP-β, two isoforms of ChREBP transcribed from different promoters, are both transcriptionally induced by glucose. However, the mechanism by which glucose increases ChREBP mRNA levels remains unclear. Here we report that hepatocyte nuclear factor 4 alpha (HNF-4α) is a key transcription factor for glucose-induced ChREBP-α and ChREBP-β expression. Ectopic HNF-4α expression increased ChREBP transcription while knockdown of HNF-4α greatly reduced ChREBP mRNA levels in liver cancer cells and mouse primary hepatocytes. HNF-4α not only directly bound to an E-box-containing region in intron 12 of the ChREBP gene, but also promoted ChREBP-β transcription by directly binding to two DR1 sites and one E-box-containing site of the ChREBP-β promoter. Moreover, HNF-4α interacted with ChREBP-α and synergistically promoted ChREBP-β transcription. Functionally, HNF-4α suppression reduced glucose-dependent ChREBP induction. Increased nuclear abundance of HNF-4α and its binding to *cis*-elements of ChREBP gene in response to glucose contributed to glucose-responsive ChREBP transcription. Taken together, our results not only revealed the novel mechanism by which HNF-4α promoted ChREBP transcription in response to glucose, but also demonstrated that ChREBP-α and HNF-4α synergistically increased ChREBP-β transcription.

ChREBP was identified as an important factor mediating the transcriptional effects of glucose on glycolytic genes such as L-type pyruvate kinase (L-PK) in hepatocytes^1^. Glucose increases ChREBP expression and activity at the transcriptional and posttranslational levels, promoting its nuclear translocation and DNA binding activity^2^,^3^. ChREBP heteromerizes with Max-like protein X (Mlx) and binds to the *cis*-element called carbohydrate response element (ChoRE) consisting of two E-box sites separated by 5 nucleotides^4^. Several genes encoding key enzymes for glycolysis and lipogenesis have been demonstrated to be direct ChREBP target genes, including L-PK, acetyl-CoA carboxylase (ACC), fatty acid synthase (FAS) and stearoyl-CoA desaturase-1 (SCD1)^1^,^5^,^6^,^7^,^8^. In addition to the canonical ChREBP isoform (ChREBP-α), the newly identified isoform ChREBP-β transcribed from a different promoter plays an important role in linking lipogenesis in adipose tissue to insulin sensitivity^9^. ChREBP-β is a target gene of ChREBP-α and the ChoRE sequences in the ChREBP-β promoter have been identified^9^,^10^.

In addition to glucose, signals such as insulin, polyunsaturated fatty acids (PUFA), lipopolysaccharide (LPS), branched-chain amino acids (BCAAs), anoxia and TGF-β can positively or negatively regulate the mRNA level of ChREBP^11^,^12^,^13^,^14^. Moreover, mRNA levels of ChREBP are altered in obese adolescents with prediabetes, early type 2 diabetes or during human cytomegalovirus (HCMV) infection^15^,^16^,^17^. We have recently reported that transcription of ChREBP can be induced by glucose or advanced glycation end products (AGEs) in cancer cells^18^,^19^. These findings all indicate that mRNA levels of ChREBP-α and ChREBP-β are dynamically regulated in different types of cells in response to various signals. ChREBP-α has been reported to be a direct target gene of thyroid hormone receptor beta (TR-β) and liver X receptor (LXR)^20^,^21^. However, LXR and TR-β may not mediate glucose-induced ChREBP transcription^21^,^22^,^23^. So far it remains unclear how glucose increases ChREBP-α and ChREBP-β transcription.

Hepatocyte nuclear factor 4 alpha (HNF-4α), a highly conserved member of the nuclear receptor superfamily, is abundant in fetal and adult liver and intestine^24^. HNF-4α can not only directly regulate expression of a large number of target genes encoding enzymes in glucose, fatty acid, cholesterol and drug metabolism in the liver, but also activate other transcription factors that in turn regulate the expression of liver-specific target genes^25^,^26^,^27^,^28^,^29^. The embryonic lethality of HNF-4α knockout mice is probably due to the requirement for HNF-4α in liver development^30^,^31^,^32^. HNF-4α is essential for morphological and functional differentiation of hepatocytes^32^. HNF-4α often binds as a homodimer to its DNA recognition sequence, the classical DR1 (AGGTCAxAGGTCA) or H4-SBM (xxxxCAAAGTCCA) site^33^. Recent study shows that, in addition to DR1 and H4-SBM sites, HNF-4α also binds to a non-perfect DR1 site which contains the E-box sequence in the proximal promoter of the murine pyruvate carboxylase gene^34^. Moreover, the findings that binding of HNF-4α to the FAS promoter is increased by glucose and HNF-4α co-immunoprecipitates with ChREBP in primary hepatocytes suggest that HNF-4α might play an important role in glucose-induced transcriptional activation of metabolic enzyme genes^35^.

Here we discovered that HNF-4α overexpression increased transcription of both human ChREBP-α and ChREBP-β whereas siRNA-mediated HNF-4α knockdown reduced ChREBP-α and ChREBP-β mRNA levels in human 293T, HepG2 cells and mouse primary hepatocytes. Further analysis revealed that HNF-4α promoted ChREBP-α and ChREBP-β transcription by directly binding to an E-box-containing sequence in intron 12 and DR1 sites in the ChREBP-β promoter, respectively. ChREBP-α cooperated with HNF-4α in promoting ChREBP-β transcription. Glucose increased HNF-4α mRNA and protein levels, nuclear abundance and its binding to *cis*-elements of ChREBP gene. Moreover, knockdown of HNF-4α greatly reduced glucose-dependent ChREBP-α and ChREBP-β induction.

## Results

### Ectopic HNF-4α expression increases mRNA levels of ChREBP-α and ChREBP-β

**Figure 1:**
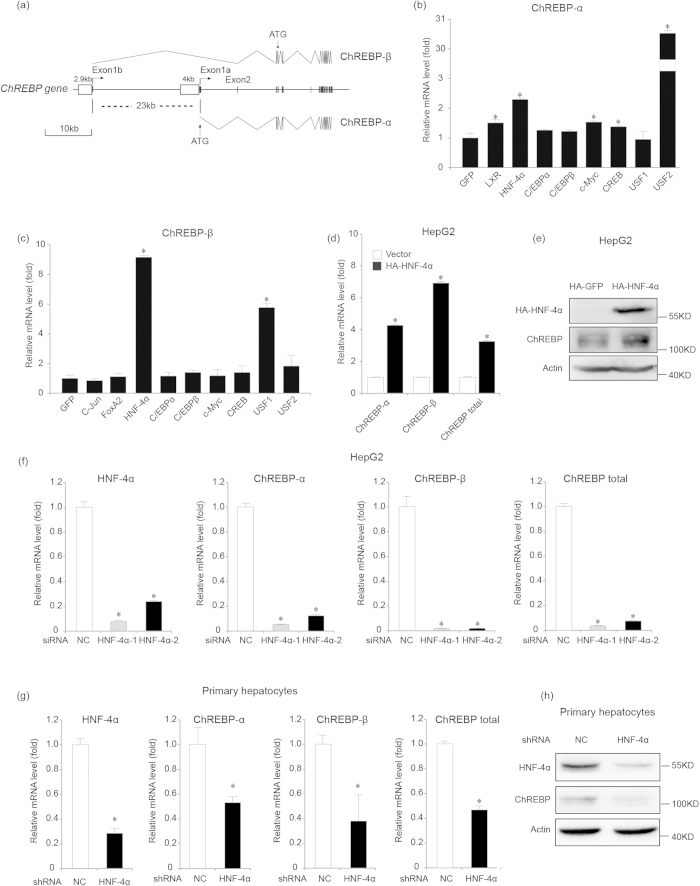
HNF-4α promotes ChREBP-α and ChREBP-β transcription. (**a**). Gene structure of human ChREBP-α and ChREBP-β with indication of splice sites and translational start sites (ATG). Note that ChREBP-α and ChREBP-β are transcribed from different promoters separated by 23 kb. (**b**). Real-time PCR analysis for mRNA levels of ChREBP-α at 48 hours after expression plasmids containing control (GFP) or other cDNAs are transfected in 293T cells. *indicates p < 0.05 when compared with the GFP-transfected sample. (**c**). Real-time PCR analysis for mRNA levels of ChREBP-β at 48 hours after expression plasmids containing control (GFP) or other cDNAs are transfected in 293T cells. *indicates p < 0.05 when compared with the GFP-transfected sample. (**d**,**e**). Real-time PCR analysis for mRNA levels of ChREBP-α, ChREBP-β and total ChREBP (**d**) and western blot analysis for endogenous ChREBP expression (**e**) at 48 hours after HA-GFP (vector) or HA-HNF-4α expression plasmids are transfected in HepG2 cells. *indicates p < 0.05 when compared with the vector-transfected sample. Tubulin serves as the loading control. (**f**).Real-time PCR analysis for mRNA levels of HNF-4α, ChREBP-α, ChREBP-β and total ChREBP at 72 hours after control (NC) or two HNF-4α siRNAs (HNF-4α-1 and HNF-4α-2) are transfected in HepG2 cells. *indicates p < 0.05 when compared with the corresponding NC-transfected sample. (**g**,**h**). Real-time PCR analysis for mRNA levels of HNF-4α, ChREBP-α, ChREBP-β and total ChREBP (**g**) and western blot analysis for endogenous ChREBP expression (**h**) at 48 hours after control (NC) or HNF-4α shRNAs are transfected in mouse primary hepatocytes. *indicates p < 0.05 when compared with the corresponding NC-transfected sample. Actin serves as the loading control.

In order to search for potential binding sites for known transcription factors in the promoter region, we analyzed the 4 kb (nucleotides −4000~−1) and 2.9 kb (nucleotides −2612~+271) DNA sequences of human ChREBP-α and ChREBP-β genes using the rvista software (http://genome.lbl.gov/vista/rvista/submit.shtml) (Fig. 1a). Position +1 represents the transcription start site for human ChREBP-α and ChREBP-β genes, respectively. The analysis revealed candidate binding sites for LXR, HNF-4α, C/EBPα, C/EBPβ, c-Myc, CREB, USF1 and USF2 in the 4 kb promoter of ChREBP-α and those for c-Jun, FoxA2, HNF-4α, C/EBPα, C/EBPβ, c-Myc, CREB, USF1 and USF2 in the 2.9 kb promoter of ChREBP-β. Next we ectopically expressed these transcription factors in 293T cells and analyzed mRNA levels of ChREBP-α and ChREBP-β using real time PCR analysis. We found that, among these transcription factors, HNF-4α promoted transcription of both ChREBP-α and ChREBP-β (Fig. 1b,c). HNF-4α seemed to be more potent in promoting ChREBP-α transcription when compared to LXR, which was reported to increase ChREBP-α mRNA levels^20^,^21^ (Fig. 1b,c). Using primers specific for ChREBP-α or ChREBP-β and those common for ChREBP-α and ChREBP-β, we found that HNF-4α increased mRNA levels of ChREBP-α, ChREBP-β and total ChREBP in 293T and liver cancer HepG2 cells (Fig. 1d, Supplementary Fig. 1a). Although transcription of ChREBP-β was more induced by HNF-4α than that of ChREBP-α, the fold of induction of total ChREBP was similar to that of ChREBP-α probably because ChREBP-α was much more abundant than ChREBP-β in these cells. Using an anti-ChREBP antibody which detected both ChREBP-α and ChREBP-β protein, we found that the protein level of ChREBP also increased after HNF-4α overexpression in 293T and HepG2 cells (Fig. 1e, Supplementary Fig. 1b). Moreover, we analyzed mRNA levels of ChREBP target genes such as FAS, ACC, L-PK and SCD1 after overexpressing HNF-4α in 293T cells. HNF-4α promoted transcription of L-PK and had a mild effect on FAS, ACC and SCD1 mRNA levels (Supplementary Fig. 1c).

In order to further investigate the effect of suppressing HNF-4α expression on ChREBP transcription, we transfected HepG2 cells with two independent siRNAs for HNF-4α and successfully suppressed HNF-4α expression (Fig. 1f). In comparison to the non-targeting control siRNA, both siRNAs for HNF-4α decreased mRNA levels of ChREBP-α, ChREBP-β and total ChREBP in HepG2 cells (Fig. 1f). We also transfected mouse primary hepatocytes with the control shRNA and shRNA for HNF-4α. When compared to the control, HNF-4α knockdown reduced ChREBP mRNA and protein levels in mouse primary hepatocytes (Fig. 1g,h).

### HNF-4α and ChREBP expression in mouse primary hepatocytes is up-regulated by glucose

**Figure 2:**
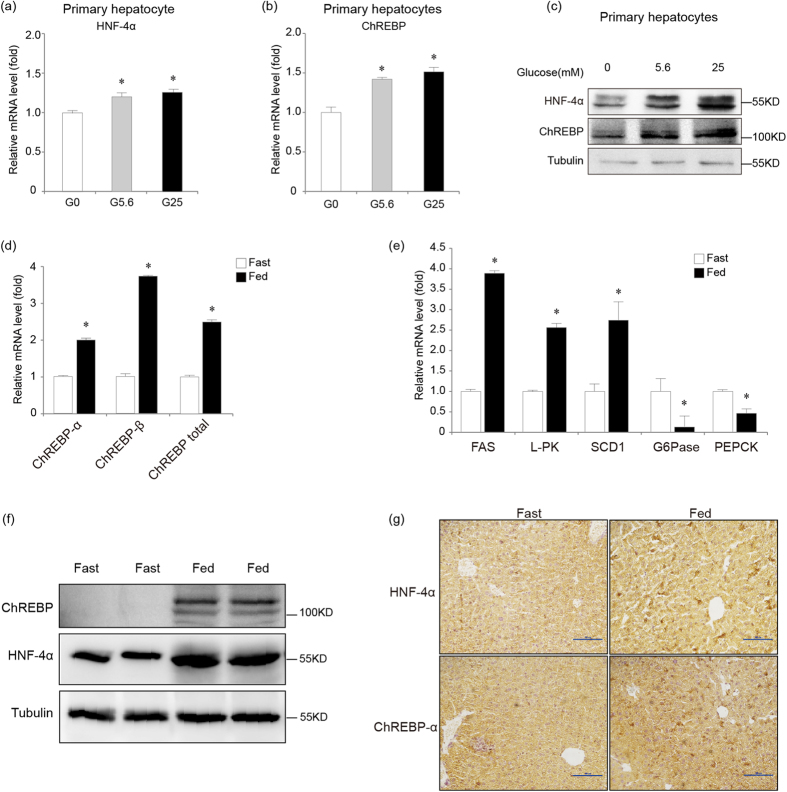
Glucose increases HNF-4α and ChREBP expression. (**a**,**b**). Real-time PCR analysis for mRNA levels of HNF-4α (A) and total ChREBP (B) in mouse primary hepatocytes treated with 0 (G0), 5.6 (G5.6) or 25 mM glucose (G25) for 18 hours. *indicates p < 0.05 when compared with the corresponding 0 mM glucose-treated sample. (**c**). Western blot analysis for endogenous ChREBP and HNF-4α protein expression in mouse primary hepatocytes treated with 0, 5.6 or 25 mM glucose for 18 hours. Tubulin serves as the loading control. (**d**). Real-time PCR analysis for mRNA levels of ChREBP-α, ChREBP-β and total ChREBP in mouse liver after being fasted or fed for 18 hours. *indicates p < 0.05. (**e**). Real-time PCR analysis for mRNA levels of FAS, L-PK, SCD1, G6Pase and PEPCK in mouse liver after being fasted or fed for 18 hours. *indicates p < 0.05. (**f**). Western blot analysis for endogenous ChREBP and HNF-4α protein expression in mouse liver after being fasted or fed for 18 hours. Tubulin serves as the loading control. (**g**). Immunohistochemistry analysis for mouse liver after being fasted or fed for 18 hours using anti-ChREBP and anti-HNF-4α antibodies. Bars represent 100 μm.

We treated mouse primary hepatocytes and human liver cancer HepG2 cells with 0 mM, 5.6 mM and 25 mM glucose for 18 hours, respectively. Real time PCR analysis showed that 25 mM glucose was more potent than 5.6 mM glucose to increase mRNA and protein levels of ChREBP-α, ChREBP-β and total ChREBP in comparison to the 0 mM glucose treatment in mouse primary hepatocytes and HepG2 cells (Fig. 2a–c, Supplementary Fig. 2a,b). Our results agree with reported findings that mRNA levels of ChREBP are regulated by glucose or glucose derived metabolites^2^,^3^.

To investigate the role of HNF-4α in regulating ChREBP expression in response to glucose *in vivo*, wild type C57Bl/6 mice were fasted or given access to food for 18 hours before mRNA levels of ChREBP in the liver were assayed. Transcription of ChREBP-α, ChREBP-β and total ChREBP was up-regulated in the fed state compared with the fasted control (Fig. 2d). Transcription of target genes activated by ChREBP such as FAS, L-PK and SCD1 were increased whereas those target genes repressed by ChREBP including G6Pase and PEPCK were down-regulated in the fed mouse liver, suggesting that the activity of ChREBP was induced by feeding (Fig. 2e). Interestingly, we observed up-regulation of both ChREBP and HNF-4α protein expression in response to feeding using western blot analysis and immunohistochemistry (Fig. 2f,g).

### HNF-4α binds the E-box-containing cis-element located in intron 12 of the ChREBP-α gene

**Figure 3:**
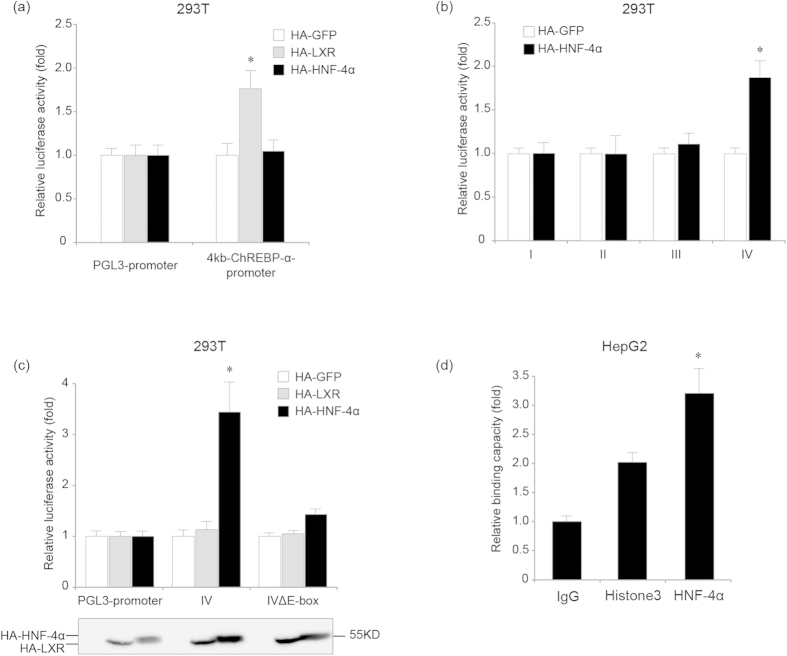
The E-box-containing *cis*-element in intron 12 mediates HNF-4α-induced ChREBP-α transcription. (**a**). Luciferase activity assay shows that HA-HNF-4α fails to increase transcriptional activity of the 4 kb ChREBP-α promoter compared with the HA-GFP-transfected sample. 24 hours after the 4 kb ChREBP-α promoter in pGL3-Basic plasmid and the HA-GFP, HA-LXR or HA-HNF-4α expression plasmid are transfected in 293T cells, luciferase activity is analyzed. *indicates p < 0.05 when compared with the HA-GFP-transfected sample. (**b**). Luciferase activity assay shows that HNF-4α enhances transcriptional activity of the pGL3-Promoter plasmid containing the 140 bp sequence in intron 12 of ChREBP-α (IV) compared with the control. I, II, III and IV are the pGL3-Promoter plasmids containing the 164 bp, 174 bp, 378 bp and 140 bp sequences located in intron 2, intron 6, intron 7 and intron 12 of ChREBP-α. 24 hours after I, II, III or IV and the HA-GFP or HA-HNF-4α expression plasmid are transfected in 293T cells, luciferase activity is analyzed. *indicates p < 0.05 when compared with the HA-GFP-transfected sample. (**c**). Luciferase activity assay shows that deletion of the E-box in IV reduced induction of the transcriptional activity of IV by HNF-4α compared with the control. 24 hours after the pGL3-Promoter plasmid, IV or IVΔE-box and the HA-GFP, HA-LXR or HA-HNF-4α expression plasmid are transfected in 293T cells, luciferase activity is analyzed. *indicates p < 0.05 when compared with the HA-GFP-transfected sample. Western blot analysis using the anti-HA antibody shows protein levels of ectopically expressed HA-HNF-4α and HA-LXR. (**d**). ChIP analysis for HepG2 cells using an anti-HNF-4α antibody, nonspecific IgG or anti-histone antibody shows that HNF-4α binds the 140 bp region in intron 12 of the ChREBP-α gene.

Next we explored the molecular mechanism by which HNF-4α promotes ChREBP-α and ChREBP-β transcription. The 4 kb promoter of ChREBP-α was predicted to contain potential binding sites for HNF-4α. Therefore, we transiently transfected 293T cells with the 4 kb ChREBP-α promoter in pGL3-Basic plasmid along with HNF-4α or control expression plasmids and analyzed luciferase activity 48 hours later. Since LXR was reported to promote ChREBP transcription^21^, it was included as a positive control. Although LXR increased the ChREBP-α promoter luciferase reporter activity, we were not able to detect any induction in luciferase reporter activity by HNF-4α overexpression in comparison to the negative control (Fig. 3a). This finding suggests that the 4 kb promoter of ChREBP-α might not contain potential binding sites for HNF-4α.

We next searched the whole human ChREBP-α gene sequence for potential binding sites for HNF-4α, including classical DR1, H4-SBM sites and the non-perfect DR1 site which contains the E-box sequence. We found four E-box-containing non-perfect DR1 sites located in intron 2, intron 6, intron 7 and intron 12 of the ChREBP-α gene. We cloned the 164 bp, 174 bp, 378 bp and 140 bp sequences containing the non-perfect DR1 sites located in intron 2, intron 6, intron 7 and intron 12 of the ChREBP-α gene from human genomic DNA and subcloned them into the pGL3-Promoter vector containing an SV40 promoter upstream of the luciferase reporter gene, respectively. We named these four pGL3-Promoter plasmids containing the 164 bp, 174 bp, 378 bp and 140 bp intronic sequences as plasmid I, II, III and IV, respectively. Insertion of DNA fragments containing potential binding sites for HNF-4α into the pGL3-Promoter vector allowed for screening for putative enhancer elements regulated by HNF-4α. We transiently transfected 293T cells with plasmids I, II, III or IV along with HNF-4α or control expression plasmids and analyzed luciferase activity. Only the luciferase activity of plasmid IV, the pGL3-Promoter plasmid containing the 140 bp sequence in intron 12, was enhanced by HNF-4α overexpression compared with the control (Fig. 3b). We transfected 293T cells with the plasmid IV along with LXR or control expression plasmids and found that LXR was not able to induce luciferase activity, suggesting that the induction in luciferase activity of plasmid IV by HNF-4α should be specific (Fig. 3c). We also deleted the E-box (CACGTG) in the 140 bp sequence in intron 12 and generated the E-box free plasmid IV. We found that loss of the E-box greatly reduced induction of the luciferase activity by HNF-4α overexpression (Fig. 3c).

In order to determine whether endogenous HNF-4α directly bound the E-box-containing *cis*-element located in intron 12 of the ChREBP-α gene, we performed ChIP analysis for HepG2 cells using an anti-HNF-4α antibody. Quantitative PCR analysis for DNA fragments encompassing the E-box-containing *cis*-element showed that HNF-4α directly bound the E-box-containing region in intron 12 of the ChREBP-α gene (Fig. 3d).

### HNF-4α binds DR1 sites located in the ChREBP-β promoter

**Figure 4:**
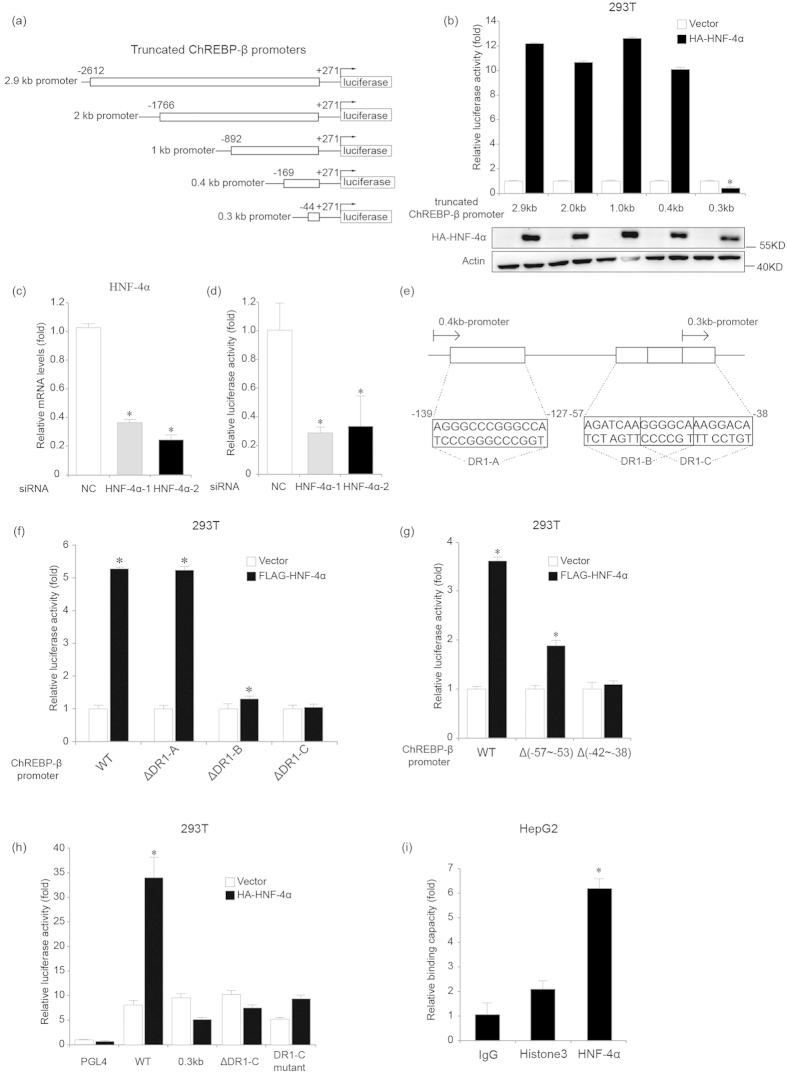
The DR1 sites in the ChREBP-β promoter mediate HNF-4α-induced ChREBP-β transcription. (**a**). Schematic diagrams of the ChREBP-β promoter fragments in the pGL4-Basic plasmid. (**b**). Luciferase activity analysis for the ChREBP-β promoter fragments in the pGL4-Basic plasmid at 24 hours after the ChREBP-β promoter fragment plasmids and empty vector or HA-HNF-4α expression plasmid are transfected in 293T cells. Western blot analysis using the anti-HA antibody shows protein levels of ectopically expressed HA-HNF-4α. Actin serves as the loading control. *indicates p < 0.05 when compared with the other four black bars. (**c**). Real-time PCR analysis for mRNA levels of HNF-4α at 72 hours after control (NC) or two HNF-4α siRNAs (HNF-4α-1 and HNF-4α-2) are transfected in 293T cells. (**d**). Luciferase activity analysis for the 2.9 kb ChREBP-β promoter in the pGL4-Basic plasmid at 72 hours after the ChREBP-β promoter plasmid and control or two HNF-4α siRNAs are transfected in 293T cells. (**e**). DNA sequences of the three DR1 sites in the nucleotides −139 to −38 region of the ChREBP-β promoter. (**f**). Luciferase activity analysis for the 2.9 kb (WT), ΔDR1-A, ΔDR1-B and ΔDR1-C ChREBP-β promoter in the pGL4-Basic plasmids at 24 hours after the ChREBP-β promoter plasmids and empty vector or FLAG-HNF-4α expression plasmid are transfected in 293T cells. (**g**). Luciferase activity analysis for the 2.9 kb (WT), Δ(−57~−53) and Δ(−42~−38) ChREBP-β promoter in the pGL4-Basic plasmids at 24 hours after the ChREBP-β promoter plasmids and empty vector or FLAG-HNF-4α expression plasmid are transfected in 293T cells. (**h**). Luciferase activity analysis for the pGL4, 2.9 kb (WT), 0.3 kb, ΔDR1-C and DR1-C mutant ChREBP-β promoter in the pGL4-Basic plasmids at 24 hours after the ChREBP-β promoter plasmids and empty vector or HA-HNF-4α expression plasmid are transfected in 293T cells. The DR1-C mutant contains 7 underlined point mutations (GCGGACTCTGAAA). **(i).** ChIP analysis for HepG2 cells using an anti-HNF-4α antibody, nonspecific IgG or anti-histone antibody shows that HNF-4α binds ChREBP-β promoter. *in C-D and F-H indicates p < 0.05 when compared with the corresponding NC or empty vector -transfected sample.

To identify possible HNF-4α-binding sites in the promoter of ChREBP-β, we cloned the 2.9 kb ChREBP-β promoter from human genomic DNA and subcloned it into the luciferase reporter vector pGL4-Basic (Fig. 4a). We transiently transfected the 2.9 kb ChREBP-β promoter in pGL4-Basic plasmid into 293T cells along with HNF-4α or control expression plasmids and analyzed luciferase activity 48 hours later. HNF-4α increased the 2.9 kb ChREBP-β promoter luciferase reporter activity in 293T cells by about 12 folds in comparison to controls (Fig. 4b). We next investigated the effect of suppressing HNF-4α expression on activity of human 2.9 kb ChREBP-β promoter. We transfected 293T cells with the 2.9 kb ChREBP-β promoter in pGL4-Basic plasmid along with control siRNAs or siRNAs for HNF-4α and found that HNF-4α knockdown decreased luciferase activity in comparison to the control (Fig. 4c,d).

In order to further determine the location of *cis*-elements for HNF-4α in the ChREBP-β promoter, we generated ChREBP-β promoter fragments from nucleotides −1766, −892, −169 and −44 to the nucleotide +271 and cloned them into the pGL4-Basic plasmid (Fig. 4a). After transfecting 293T cells with pGL4-Basic plasmids containing different fragments of the ChREBP-β promoter along with control or HNF-4α expression plasmid, we assessed for their transcriptional activity. The transcriptional activity of the 2.9 kb, 2.0 kb, 1.0 kb and 0.4 kb ChREBP-β promoter fragments was 10- to 12- fold increased by HNF-4α (Fig. 4b). However, HNF-4α could not promote the luciferase activity of the 0.3 kb ChREBP-β promoter fragment, suggesting that *cis*-elements for HNF-4α were located in the nucleotides −169~−44 region of the ChREBP-β promoter (Fig. 4b). We found three DR1 sites within the nucleotides −139~−38 region of the ChREBP-β promoter and named them DR1-A (−139~−127), DR1-B (−57~−45) and DR1-C (−50~−38) (Fig. 4e). We generated 2.9 kb ChREBP-β promoter fragments lacking the 13 bp DR1-A, DR1-B or DR1-C site and named them ΔDR1-A, ΔDR1-B or ΔDR1-C. We transfected 293T cells with pGL4-Basic plasmids containing the 2.9 kb ChREBP-β promoter fragments or ΔDR1-A, ΔDR1-B, ΔDR1-C along with the control or HNF-4α expression plasmid and found that the DR1-B or DR1-C deletion greatly reduced the induction of luciferase activity of the ChREBP-β promoter by HNF-4α (Fig. 4f). Since DR1-B and DR1-C sites are overlapped by 6 bp (−50~−45), deletion of the whole 13 bp DR1-B or DR1-C site might affect the integrity of the overlapped DR1-C or DR1-B site. Therefore, we generated 2.9 kb ChREBP-β promoter fragments lacking the −57~−53 region of DR1-B or −42~−38 region of DR1-C and named them Δ(−57~−53) or Δ(−42~−38). After transfecting 293T cells with pGL4-Basic plasmids containing the 2.9 kb ChREBP-β promoter fragments or Δ(−57~−53) or Δ(−42~−38) along with the control or HNF-4α expression plasmid, we found that deletion of the −57~−53 region or the −42~−38 region reduced or abolished the induction of luciferase activity of the ChREBP-β promoter by HNF-4α, respectively (Fig. 4g). In addition to deletion, we have generated a 2.9 kb ChREBP-β promoter fragment with the mutated DR1-C site and found that both deletion and 7-bp mutation of DR1-C abolished the induction of luciferase activity of the ChREBP-β promoter by HNF-4α (Fig. 4h). Our findings suggest that both DR1-B and DR1-C sites are responsible for HNF-4α-induced ChREBP-β transcription.

In order to determine whether endogenous HNF-4α directly bound DR1-B and DR1-C sites of the ChREBP-β promoter, we performed ChIP analysis for HepG2 cells using an anti- HNF-4α antibody. Quantitative PCR analysis for DNA fragments encompassing the nucleotides −86~−37 region showed that HNF-4α directly bound this region of ChREBP-β promoter (Fig. 4i).

### ChREBP-α increases ChREBP-β transcription *via* a ChoRE in the human ChREBP-β promoter

**Figure 5:**
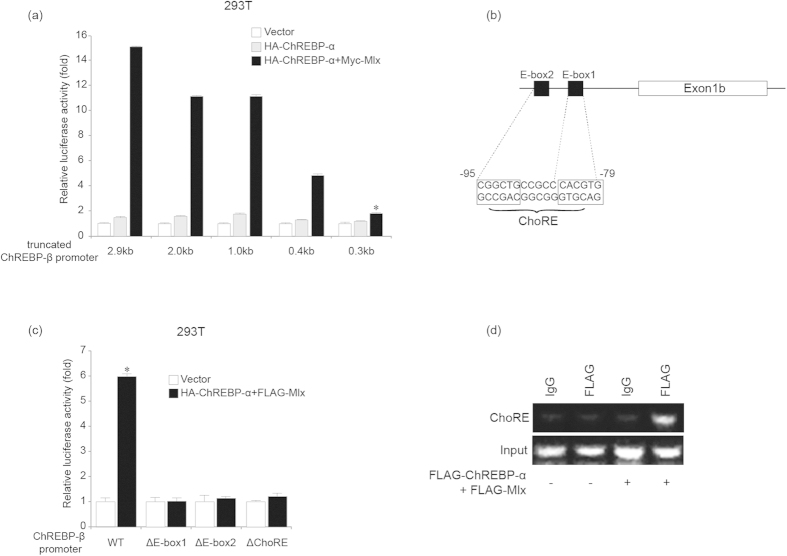
ChREBP-α increases ChREBP-β transcription by binding to the ChoRE site in the human ChREBP-β promoter. **(a).** Luciferase activity analysis for the 2.9 kb, 2.0 kb, 1.0 kb, 0.4 kb and 0.3 kb ChREBP-β promoter fragments in the pGL4-Basic plasmid at 24 hours after the ChREBP-β promoter fragment plasmids and empty vector or HA-ChREBP-α or HA-ChREBP-α and Myc-Mlx expression plasmid are transfected in 293T cells. *indicates p < 0.05 when compared with the other four black bars. **(b).** DNA sequences of the ChoRE site in the nucleotides −95 to −79 region of the ChREBP-β promoter. **(c).** Luciferase activity analysis for the 2.9 kb (WT), ΔE-box1, ΔE-box2, and ΔChoRE ChREBP-β promoter in the pGL4-Basic plasmids at 24 hours after the ChREBP-β promoter plasmids and empty vector or HA-ChREBP-α and FLAG-Mlx expression plasmid are transfected in 293T cells. *indicates p < 0.05 when compared with the corresponding empty vector-transfected sample. **(d).** ChIP analysis for 293T cells transfected with empty vector or FLAG-ChREBP-α and FLAG-Mlx expression plasmid using an anti-FLAG antibody or nonspecific IgG.

ChREBP-α and Mlx have been reported to promote transcription of ChREBP-β and the ChoRE sites have been identified in the ChREBP-β promoter^9^,^10^. We transfected 293T cells with pGL4-Basic plasmids containing different fragments of the ChREBP-β promoter along with control, ChREBP-α or ChREBP-α and Mlx expression plasmids and assessed for their transcriptional activity. As reported, co-expression of both ChREBP-α and Mlx was more potent in increasing transcription of the ChREBP-β promoter than ChREBP-α alone (Fig. 5a). The transcriptional activity of the 2.9 kb, 2.0 kb, 1.0 kb and 0.4 kb ChREBP-β promoter fragments was about 15-, 11-, 11- and 5- fold increased by ChREBP-α and Mlx (Fig. 5a). However, ChREBP-α could hardly promote the luciferase activity of the 0.3 kb ChREBP-β promoter fragment, suggesting that *cis*-elements for ChREBP-α might be located in the nucleotides −169~−44 region of the ChREBP-β promoter (Fig. 5a). As reported^10^, there is a ChoRE site (−95~−79) containing E-box 1 (−84~−79) and E-box 2 (−95~−90) separated by 5 nucleotides in this region (Fig. 5b). After transfecting 293T cells with pGL4-Basic plasmids containing the 2.9 kb ChREBP-β promoter fragment or 2.9 kb ChREBP-β promoter fragments lacking E-box 1, E-box 2 or the ChoRE along with the control or ChREBP-α and Mlx expression plasmids, we found that deleting either of the two E-boxes or the ChoRE abolished the induction of luciferase activity of the ChREBP-β promoter by ChREBP-α and Mlx (Fig. 5c). These findings suggest that the ChoRE (−95~−79) is responsible for ChREBP-α and Mlx-induced ChREBP-β transcription.

In order to determine whether ChREBP-α and Mlx directly bind the ChoRE site in the ChREBP-β promoter, we performed ChIP analysis for 293T cells transfected with either control plasmid or Flag-tagged ChREBP-α and Mlx expression plasmids using an anti-Flag antibody or nonspecific IgG. PCR analysis for DNA fragments encompassing the nucleotides −306~−60 region showed that Flag-tagged ChREBP-α and Mlx directly bind this region of ChREBP-β promoter (Fig. 5d).

### HNF-4α and ChREBP-α/Mlx additively promote ChREBP-β transcription

**Figure 6:**
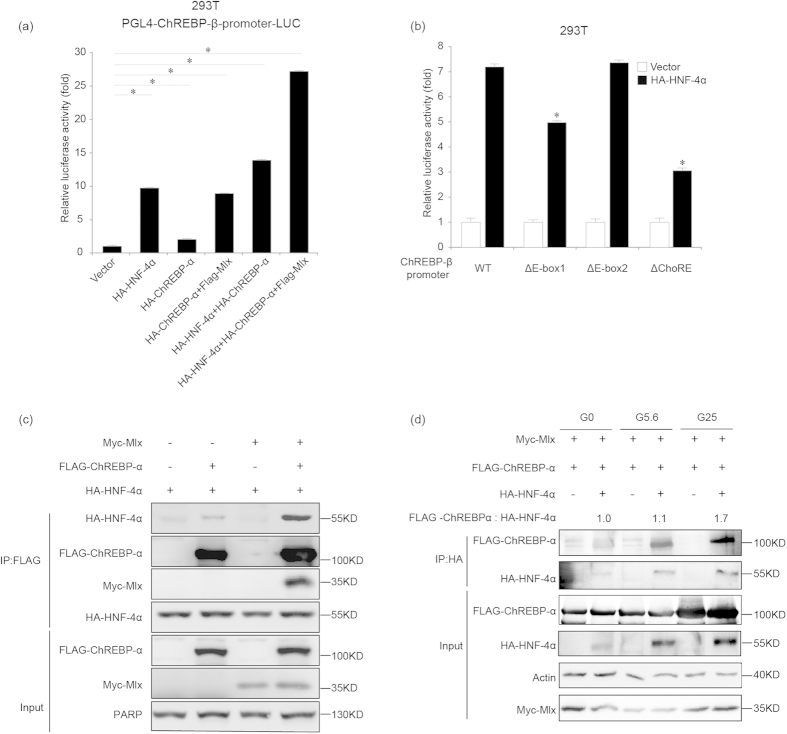
HNF-4α and ChREBP-α additively promote ChREBP-β transcription. **(a).** Luciferase activity analysis for the 2.9 kb ChREBP-β promoter in the pGL4-Basic plasmid at 24 hours after the ChREBP-β promoter plasmid and empty vector or HA-HNF-4α or HA-ChREBP-α or HA-ChREBP-α and FLAG-Mlx or HA-HNF-4α and HA-ChREBP-α or HA-HNF-4α, HA-ChREBP-α and FLAG-Mlx expression plasmids are transfected in 293T cells. *indicates p < 0.05. **(b).** Luciferase activity analysis for the 2.9 kb (WT), ΔE-box1, ΔE-box2, and ΔChoRE ChREBP-β promoter in the pGL4-Basic plasmids at 24 hours after the ChREBP-β promoter plasmids and empty vector or HA-HNF-4α expression plasmid are transfected in 293T cells. *indicates p < 0.05 when compared with the empty vector-transfected sample. **(c).** Co-IP analysis for ectopically expressed ChREBP-α, HNF-4α and Mlx at 48 hours after transfection in 293T cells. Tubulin serves as the loading control. **(d).** Co-IP analysis for ectopically expressed ChREBP-α, HNF-4α and Mlx in 293T cells treated with 0 (G0), 5.6 (G5.6) or 25 mM glucose (G25) for 18 hours. Actin serves as the loading control. The intensity of bands has been measured and the ratio between immunoprecipitated FLAG-ChREBP-α and HA-HNF-4α is shown as fold of induction at G5.6 or G25 compared with G0.

We next examined whether HNF-4α and ChREBP-α have an additive effect in promoting ChREBP-β transcription. After transfecting 293T cells with pGL4-Basic plasmids containing 2.9 kb ChREBP-β promoter along with control, HNF-4α, ChREBP-α or different combination of HNF-4α, ChREBP-α and Mlx expression plasmids, we assessed for their transcriptional activity. ChREBP-α alone increased ChREBP-β transcription by less than 2 folds, which was consistent with previous findings (Fig. 6a). The transcriptional activity of the 2.9 kb ChREBP-β promoter was about 10-fold increased by HNF-4α or ChREBP-α and Mlx (Fig. 6a). HNF-4α in combination with ChREBP-α and Mlx increased ChREBP-β transcription by more than 25 folds, suggesting that HNF-4α and ChREBP-α/Mlx cooperatively promoted ChREBP-β transcription (Fig. 6a). Deletion of the E-box 1 (−84~−79) or the ChoRE, but not the E-box 2 (−95~−90) reduced the induction of luciferase activity of the ChREBP-β promoter by HNF-4α, suggesting that the E-box 1 (−84~−79 bp) contributes to HNF-4α-induced ChREBP-β transcription. (Fig. 6b).

It has been reported that HNF-4α co-immunoprecipitates with ChREBP in primary hepatocytes^32^. We co-transfected FLAG-ChREBP-α and HA-HNF-4α in 293T cells and found that HA-HNF-4α co-immunoprecipitated with FLAG-ChREBP-α (Fig. 6c). Ectopic expression of Mlx promoted interaction between HA-HNF-4α and FLAG-ChREBP-α (Fig. 6c). We next investigated whether glucose changed the interaction between HA-HNF-4α and FLAG-ChREBP-α using co-immunoprecipitation analysis. When glucose concentration changed from 0 mM to 5.6 mM and 25 mM, increasing amount of HA-HNF-4α and FLAG-ChREBP-α protein was immunoprecipitated (Fig. 6d). When we quantified the amount of HA-HNF-4α and FLAG-ChREBP-α present in the immunoprecipitates, we found that the ratio between the amount of HA-HNF-4α and FLAG-ChREBP-α protein increased, suggesting that the interaction intensity between HA-HNF-4α and FLAG-ChREBP-α was induced by glucose (Fig. 6d).

### HNF-4α contributes to glucose-induced ChREBP-α and ChREBP-β transcription

**Figure 7:**
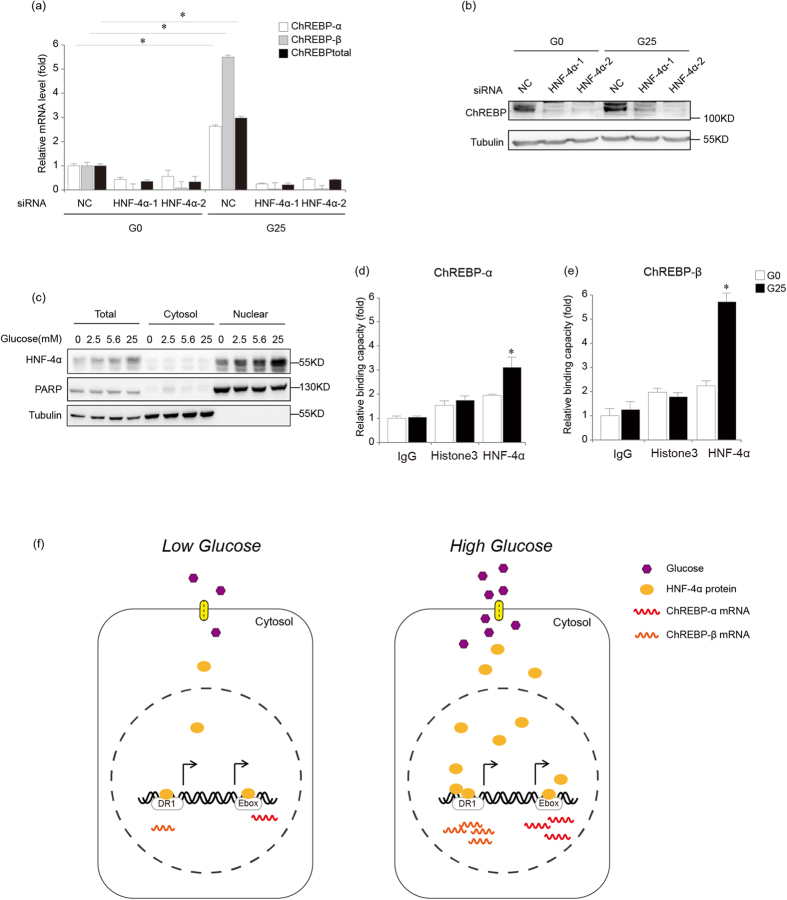
Glucose promotes ChREBP transcription through increasing HNF-4α nuclear abundance and binding to the *cis*-elements in ChREBP-α and ChREBP-β. **(a).** Real-time PCR analysis for mRNA levels of ChREBP-α, ChREBP-β and total ChREBP in HepG2 cells treated with 0 or 25 mM glucose for 18 hours after control (NC) or two HNF-4α siRNAs (HNF-4α-1 and HNF-4α-2) are transfected. *indicates p < 0.05. **(b).** Western blot analysis for endogenous ChREBP expression in HepG2 cells treated with 0 or 25 mM glucose for 18 hours after control (NC) or two HNF-4α siRNAs (HNF-4α-1 and HNF-4α-2) are transfected. Tubulin serves as the loading control. **(c).** Western blot analysis for subcellular localization of endogenous HNF-4α in HepG2 cells after 0, 2.5, 5.6 and 25 mM glucose treatment for 18 hours. PARP and tubulin serve as loading controls and cell fractionation controls. (**d**,**e**). ChIP analysis for HepG2 cells using an anti-HNF-4α antibody, nonspecific IgG or anti-histone antibody shows that binding capacity of HNF-4α for ChREBP-α (**d**) and ChREBP-β (**e**) increases under the 25 mM glucose condition (G25) when compared to the 0 mM glucose condition (G0). **(f).** Diagram showing that glucose promotes ChREBP transcription by increasing HNF-4α nuclear localization and binding to the *cis*-elements in ChREBP-α and ChREBP-β.

In order to further investigate the effect of suppressing HNF-4α expression on glucose-induced ChREBP transcription, we transfected HepG2 cells with two independent siRNAs for HNF-4α which successfully suppressed HNF-4α expression and treated cells with 0 mM and 25 mM glucose for 18 hours. In comparison to the non-targeting control siRNA, both siRNAs for HNF-4α decreased glucose-induced transcription of ChREBP-α, ChREBP-β and total ChREBP in HepG2 cells (Fig. 7a). Using the anti-ChREBP antibody which detected both ChREBP-α and ChREBP-β protein, we found that HNF-4α knockdown reduced the protein level of ChREBP induced by 25 mM glucose treatment in HepG2 cells (Fig. 7b). These findings suggest that HNF-4α contributes to glucose-induced ChREBP-α and ChREBP-β transcription.

Next we investigated the mechanism by which HNF-4α mediated the glucose-responsive effect of ChREBP transcription by studying whether and how glucose regulated subcellular localization and DNA binding capacity of HNF-4α. HepG2 cells were treated with 0 mM, 2.5mM, 5.6mM and 25 mM glucose for 18 hours before nuclear and cytoplasmic fractionation. Endogenous HNF-4α in HepG2 cells displayed increased total protein expression and nuclear abundance in response to glucose in a dose dependent manner (Fig. 7c). In order to investigate whether glucose increased HNF-4α binding to the *cis*-elements in ChREBP-α and ChREBP-β, we performed ChIP analysis for HepG2 cells treated with 0 mM and 25 mM glucose for 18 hours. Quantitative PCR analysis for DNA fragments encompassing the E-box-containing *cis*-element in ChREBP-α and the nucleotides −86~−37 region in ChREBP-β showed that glucose promoted endogenous HNF-4α binding to ChREBP-α and ChREBP-β, respectively (Fig. 7d,e). These results show that HNF-4α mediates the glucose-responsive effect of ChREBP transcription by increasing its nuclear abundance and binding to *cis*-elements in ChREBP-α and ChREBP-β (Fig. 7f).

## Discussion

Transcription of ChREBP-α and ChREBP-β from two promoters is dynamically regulated by various signals including glucose. However, the molecular mechanism triggering glucose-mediated regulation of ChREBP-α and ChREBP-β transcription remains unclear. Here we have identified the new role and mechanism by which HNF-4α promotes ChREBP-α and ChREBP-β transcription in response to glucose, revealed the molecular mechanism by which HNF-4α regulates ChREBP-α and ChREBP-β transcription, and found that ChREBP-α cooperates with HNF-4α in promoting ChREBP-β transcription.

Although much research has been carried out in determining the post-translational control of ChREBP by glucose, only a few studies have addressed the regulation of ChREBP by glucose at the transcriptional level. ChREBP is increased at the mRNA level in liver in response to high carbohydrate diet but not high fat diet^36^. Two nuclear receptors playing important roles in energy homeostasis, namely LXR and TR, have been shown to regulate ChREBP at the transcriptional level in liver^20^,^21^,^37^. Fasting and refeeding experiments have shown that ChREBP expression increases to similar levels in refed livers from wild type and TR-β null mice, suggesting that TR-β might not play a role in the nutritional regulation of ChREBP^21^. LXRα can function as a glucose-sensor and convert glucose into triglyceride by inducing lipogenic genes directly or indirectly *via* ChREBP and SREBP-1c^23^. However, ChREBP expression, its nuclear translocation and the induction of its target genes were not altered by high carbohydrate diet in liver of LXRα/β null mice, suggesting that LXR is not responsible for the effect of glucose on ChREBP^22^. Therefore, LXR and TR can promote ChREBP transcription in liver but they do not mediate glucose-induced ChREBP transcription. Here we have not only revealed the molecular mechanism by which HNF-4α promotes ChREBP-α and ChREBP-β transcription, but also have shown that HNF-4α knockdown reduced the induction of the mRNA and protein expression of ChREBP by glucose in HepG2 cells and mouse primary hepatocytes. Our results suggest that HNF-4α plays an important role in promoting ChREBP-α and ChREBP-β transcription in response to glucose. Moreover, we have revealed that glucose increases HNF-4α mRNA and protein levels, the nuclear abundance of HNF-4α and its binding to the intron of ChREBP-α or the promoter of ChREBP-β. Therefore, our findings have demonstrated that HNF-4α promotes ChREBP-α and ChREBP-β transcription in response to glucose. Glucose-induced endogenous HNF-4α binding to ChREBP-α and ChREBP-β could be due to higher levels of nuclear HNF-4α protein in response to glucose (Fig. 7c–e). Therefore, it is hard to conclude whether glucose promotes DNA binding capacity of HNF-4α. In addition, we have also noticed that USF2 and USF1 increase mRNA levels of ChREBP-α and ChREBP-β, respectively (Fig. 1b,c). It will be intriguing to find out whether USF2 and USF1 regulate transcription of ChREBP-α and ChREBP-β in response to glucose.

HNF-4α is a key transcription factor regulating hepatocyte differentiation and function^32^. HNF-4α can regulate the expression of many liver-specific target genes^25^,^26^,^27^,^28^,^29^. ChREBP-α and ChREBP-β are highly expressed in liver and our findings of ChREBP-α and ChREBP-β being HNF-4α target genes provide a possible explanation for their liver-enriched expression. HNF-4α promotes ChREBP-α and ChREBP-β transcription *via* different mechanisms. HNF-4α directly binds DR1 sites in the ChREBP-β promoter and regulates its transcription. However, the 4 kb of ChREBP promoter is not responsible for HNF-4α-induced ChREBP-α transcription. Instead, we have found that HNF-4α, but not LXR, directly binds the E-box-containing region in intron 12 of the ChREBP-α gene. This intronic sequence probably functions as an enhancer and cooperates with ChREBP-α promoter in regulating its transcription. Moreover, ChREBP-α and ChREBP-β genes share intron 12 and the E-box-containing region in intron 12 might also function as an enhancer in regulating ChREBP-β transcription.

The interaction between ChREBP and HNF-4α has been reported^38^. The glycolytic enzyme L-PK is a target gene for both ChREBP and HNF-4α^1^,^39^. Transcriptional complex containing ChREBP, HNF-4α and the co-activator CBP is necessary for the glucose-mediated induction of the L-PK gene^38^. Here we have identified a transcriptional complex containing ChREBP-α, HNF-4α and Mlx which bind to the ChoRE of the human ChREBP-β promoter and additively increases ChREBP-β transcription. Moreover, we have found that glucose might increase the interaction between HNF-4α and ChREBP-α.

## Materials and Methods

### Plasmids

We cloned the 4kb human ChREBP-α (−4000 bp~−1 bp) and 2.9kb (−2612 bp~+271 bp) human ChREBP-β promoter from human genomic DNA and subcloned them into pGL3-Basic and pGL4-Basic vector (Promega, USA), respectively. Position +1 is the transcription start site. We cloned the 164 bp, 174 bp, 378 bp and 140 bp DNA fragments containing the non-perfect DR1 sites located in intron 2, intron 6, intron 7 and intron 12 from human genomic DNA and subcloned them into the pGL3-Promoter vector (Promega, USA). The C/EBPα and c-Jun plasmids were provided by Dr. Zhaoyuan Hou and Dr. Man Mohan of Shanghai Jiao Tong University School of Medicine. The FoxA2 plasmid was provided by Dr. Jianguo Song of Chinese Academy of Sciences. The HA-HNF-4α and LXR plasmids were provided by Dr. Xiaoling Li of National Institute of Environmental Health Sciences in USA. The USF1 and USF2 plasmids were provided by Dr. Jingwen Liu of VA Palo Alto Health Care System in USA.

### Reagents

The following reagents were used: Dulbecco’s Modified Eagle’s Medium (DMEM) (Hyclone, USA), Opti-MEM and no glucose DMEM (Invitrogen, USA), Fetal bovine serum (FBS) (Biochrom, Germany), Triton X-100 and Nonidet P40 (NP-40) (Sigma, USA), anti-ChREBP (Novus Biologicals, USA), anti-HNF-4α (Absci, USA), anti-actin (Cell Signaling Technology, USA), anti-FLAG (Sigma, USA), anti-HA and anti-Myc (Santa Cruz Biotechnology, USA) antibody. All other chemicals were analytical grade.

### Mice

Six 6–8 week-old C57BL/6 wild type mice were divided into two groups. One group of three mice were fasted for 18 hours while the other group of three mice were fed with a normal diet for 18 hours before being sacrificed. Liver tissues were removed for further study. All protocols were approved by the Shanghai Jiao Tong University School of Medicine Animal Care and Use Committee. All experiments were carried out in accordance with the approved guidelines.

### Primary hepatocyte isolation, culture and treatment

Primary hepatocytes were prepared from male C57/BL6 mice using the collagenase (Gibco, USA) perfusion method^40^ and plated in six-well tissue culture plates or 10 cm dish (Corning, USA) at a density of 1 × 10^6^ cells /ml in hepatocyte medium (ScienCell, USA) supplemented with 5% FBS, 1% hepatocyte growth supplement and 1% penicillin/streptomycin antibiotic mix. After primary hepatocytes attached, the medium was replaced by DMEM supplemented with 10% FBS, 1% L-glutamine and 1% penicillin/streptomycin antibiotic mix. Primary hepatocytes were cultured in DMEM containing 5.6 mM glucose for 24 hours before 0 mM, 5.6 mM or 25 mM glucose treatment.

### Cell lines and glucose treatment

293T human embryonic kidney cells and HepG2 human hepatocellular carcinoma cells were cultured in DMEM supplemented with 10% FBS, 1% L-glutamine and 1% penicillin/streptomycin antibiotic mix. Cells at 80% confluency were transfected with plasmids or siRNAs. 48 hours after transfection, cells were harvested for subsequent experiments. For the glucose treatment experiments, cells were cultured in DMEM containing 5.6 mM glucose for 4–12 hours before being treated with 0 mM, 2.5 mM, 5.6 mM or 25 mM glucose medium for 18 hours.

### Western blot analysis

Cells were washed twice with PBS and lysed in 1% Triton-X-100 lysis buffer (50 mM Tris, 150 mM NaCl, 0.5% deoxycholate, 0.1% SDS and 1% Triton X-100) with protease inhibitors (Roche, Switzerland) at 4 °C for 30 minutes. Western blot analysis was performed as described previously^19^.

### Real time PCR analysis

Total RNA was isolated with Trizol and reverse-transcribed into cDNA using the PrimeScript RT Master Mix kit (Takara Bio, Japan). The expression of specific genes was quantitated by real time PCR using SYBR Premix Ex Taq kit (Takara Bio, Japan) on an ABI 7500HT machine (Applied Biosystems, USA) with β-actin (human) or 18sRNA (mouse) as internal controls.

### SiRNA transfection

The siRNA oligonucleotides were purchased from Jima pharmaceutical company (Shanghai, China). The control siRNA sequences were 5′-UUCUCCGAACGUGUCACGUTT-3′. The HNF-4α siRNA sequences were 5′-UCUUGUCUUUGUCCACCACTT-3′and 5′-AAUGUAGUCAUUGCCUAGGTT-3’. The siRNA oligonucleotides were transfected using Lipofectamine^TM^ RNAiMAX (Invitrogen, USA) according to the manufacturer’s protocol.

### Luciferase reporter assay

293T cells were transfected with 0.5 μg of the reporter plasmid, 0.1 μg of the β-gal plasmid and 1 μg of either pcDNA3-HNF-4α or empty vector. 24 hours later, cells were harvested and assayed using the luciferase reporter assay system (Promega, USA) and Beta-Gal Assay Kit (Beyotime, China) according to the manufacturers’ instructions.

### Chromatin immunoprecipitation (ChIP)

ChIP was performed using an EZ-ChIP kit (Millipore, USA) according to the manufacturer’s protocol. Sequences of primers used in PCR for the E-box-containing *cis*-element in intron 12 of the human ChREBP-α gene are 5′-GGGCTGCAACATGAGTCATTGGGGT-3′ (forward) and 5′-CTGGGCCCAGGTCAAGGAGCTGC-3′ (reverse). Sequences of primers used in PCR for the region containing DR1 sites in the human ChREBP-β promoter are 5′-CCCACGTGCTAAGGAGAGAA-3′ (forward) and 5′-GTGTCCTTTGCCCCTTGATC-3′ (reverse). Sequences of primers used in PCR for the region containing the ChoRE site in the human ChREBP-β promoter 5′-GCGTCTTTCTCTGCCCAC-3′ (forward) and 5′-TGCCTCCTTCTCTCCTTAGC-3′ (reverse).

### Co-immunoprecipitation

Cells were freshly lysed in the lysis buffer (1 mM EDTA, 40 mM Tris-HCl, pH 8, 100 mM NaCl, 0.5% NP-40, 1% TritonX-100), and incubated with primary antibodies at 4 °C overnight, followed by an additional 2-hour incubation with protein A/G-agarose beads (Santa Cruz Biotechnology, USA) at 4 °C. The beads were washed with the lysis buffer and boiled in 2× SDS protein loading buffer. Western blot analysis was performed after immunoprecipitation. For quantification, NIH image software was used.

### Nuclear and cytoplasmic protein extraction

All the following steps were performed, and all buffers contained the EDTA-free protease inhibitor cocktail (Roche, Switzerland). After PBS rinses, cells were centrifuged at 2000 × g for 5 min at 4 degree. Cell pellets were fractionated as described previously^19^.

### Histology and immunohistochemistry

Livers were fixed in 4% paraformaldehyde overnight and embedded in paraffin. Immunohistochemistry was performed according to standard procedures. The following primary and secondary antibodies were used: anti-ChREBP (Novus Biologicals, USA) and anti-HNF-4α (Absci, USA) antibodies; biotinylated anti-rabbit secondary antibodies (Dako, Denmark); streptavidin-HRP (Invitrogen, USA).

### ShRNA transfection

The shRNA for mouse HNF-4α (5′-AGGCTGTTGGATGAATTGAGG-3′) was obtained from Open Biosystems (Huntsville, AL, USA) and then subcloned into the lentiviral vector PLKO. The shRNA were transfected into 293T cells with the packaging plasmids PMD2G and psPAX2 using Lipofectamine 2000 (Invitrogen Life Technologies, USA) according to the manufacturer′s instructions. Viral supernatant was collected after 48 hours. 1 ml viral supernatant and 1 ml fresh medium were added to primary hepatocytes per well in six-well plates, and the medium was replaced by fresh medium after 24 hours.

### Statistics and data analysis

Experiments were performed at least three times independently and one representative experiment was shown.

## Additional Information

**How to cite this article**: Meng, J. *et al.* Identification of HNF-4α as a key transcription factor to promote ChREBP expression in response to glucose. *Sci. Rep.* **6**, 23944; doi: 10.1038/srep23944 (2016).

## Supplementary Material

Supplementary Information
