# Supplementary material for: Identification of HNF-4α as a key transcription factor to promote ChREBP expression in response to glucose
Source: Sci Rep. 2016 Mar 31;6:23944. doi: 10.1038/srep23944 (PMC4814918; doi:10.1038/srep23944)
Supplement: Supplementary Information [file srep23944-s1.pdf]

Identification of HNF-4α as a key transcription factor to promote ChREBP expression in response to glucose

Jian Meng[1,#], Ming Feng[1,#], Weibing Dong[2], Yemin Zhu[1], Yakui Li[1], Ping Zhang[1], Lifang Wu[1], Minle Li[1], Ying Lu[1], Hanbei Chen[3], Xing Liu[4], Yan Lu[4], Haipeng Sun[2], Xuemei Tong[1,*]

[1]Department of Biochemistry and Molecular Cell Biology, Shanghai Key Laboratory for Tumor Microenvironment and Inflammation, Shanghai Jiao Tong University School of Medicine, 280 S. Chongqing Road, Shanghai, 200025, China.

[2]Department of Pathophysiology, Key Laboratory of Cell Differentiation and Apoptosis of Chinese Ministry of Education, Shanghai Jiao Tong University School of Medicine, 280 S. Chongqing Road, Shanghai, 200025, China.

[3]Department of Endocrinology, Xinhua Hospital, Shanghai Jiao Tong University School of Medicine, 1665, Kong Jiang Road, Shanghai, 200092, China.

[4]Department of Endocrinology and Metabolism, Ruijin Hospital, Shanghai Jiao Tong University School of Medicine, 197 , Rui-Jin 2nd Road, Shanghai, 200025, China.

[#]These authors contributed equally to this work.

[*]Correspondence and requests for materials should be addressed to Xuemei Tong

(email: xuemeitong@shsmu.edu.cn)

Supplementary Figure legends

Supplementary Figure 1. HNF-4α promotes ChREBP-α and ChREBP-β transcription in 293T cells.

(a). Real-time PCR analysis for mRNA levels of ChREBP-α, ChREBP-β and total ChREBP at 48 hours after expression plasmids containing control (GFP) or HA- HNF-4α are transfected. * indicates $p<0.05$ when compared with the GFP-transfected sample.

(b). Western blot analysis for endogenous ChREBP protein expression at 48 hours after HA-GFP or HA-HNF-4α expression plasmids are transfected. Tubulin serves as the loading control.

(c). Real-time PCR analysis for mRNA levels of FAS, ACC, L-PK and SCD1 at 48 hours after empty vector or HA-HNF-4α expression plasmid is transfected. * indicates $p<0.05$ when compared with the corresponding empty vector-transfected sample.

Supplementary Figure 2. Glucose increases HNF-4α and ChREBP expression in HepG2 cells.

(a). Real-time PCR analysis for mRNA levels of ChREBP-α, ChREBP-β and total ChREBP in HepG2 cells treated with 0 (G0) or 25 mM glucose (G25) for 18 hours. * indicates $p<0.05$ when compared with the corresponding 0 mM glucose-treated sample.

(b). Western blot analysis for endogenous HNF-4α and ChREBP in HepG2 cells after being treated with 0, 2.5, 5.6 and 25 mM glucose for 18 hours. Actin serves as the loading control.

Supplementary Table 1. List of primers for real time PCR analysis.

Supplementary figure 1

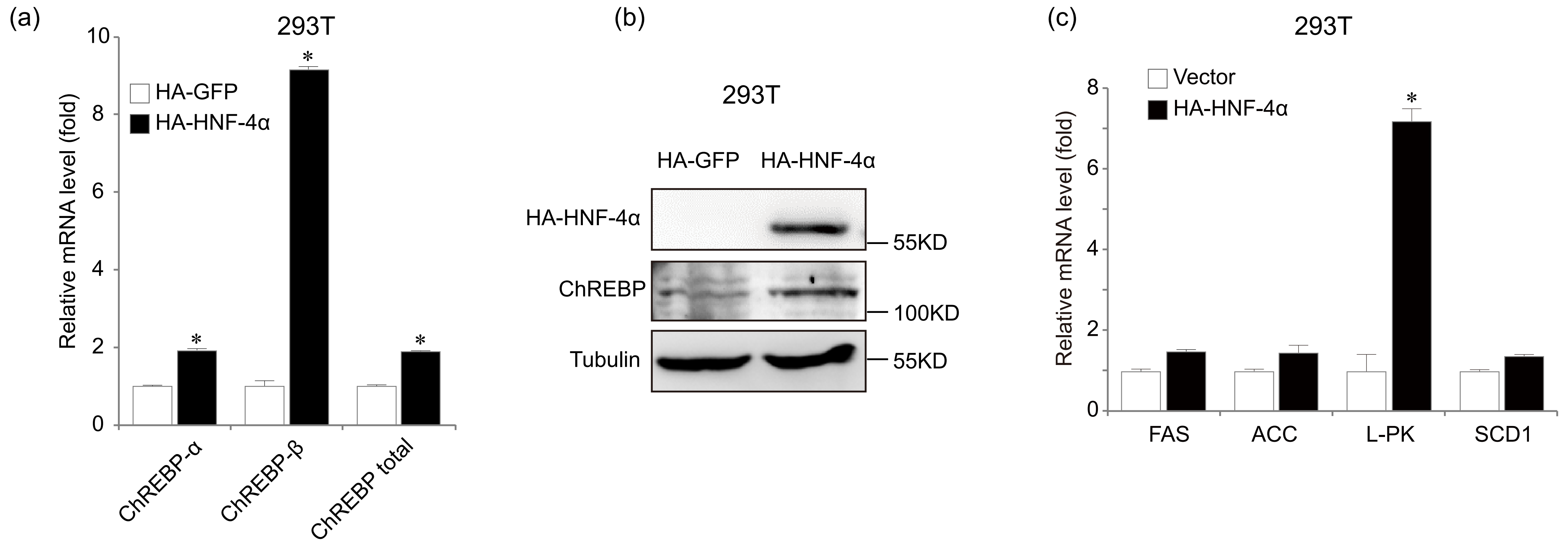

Supplementary figure 2

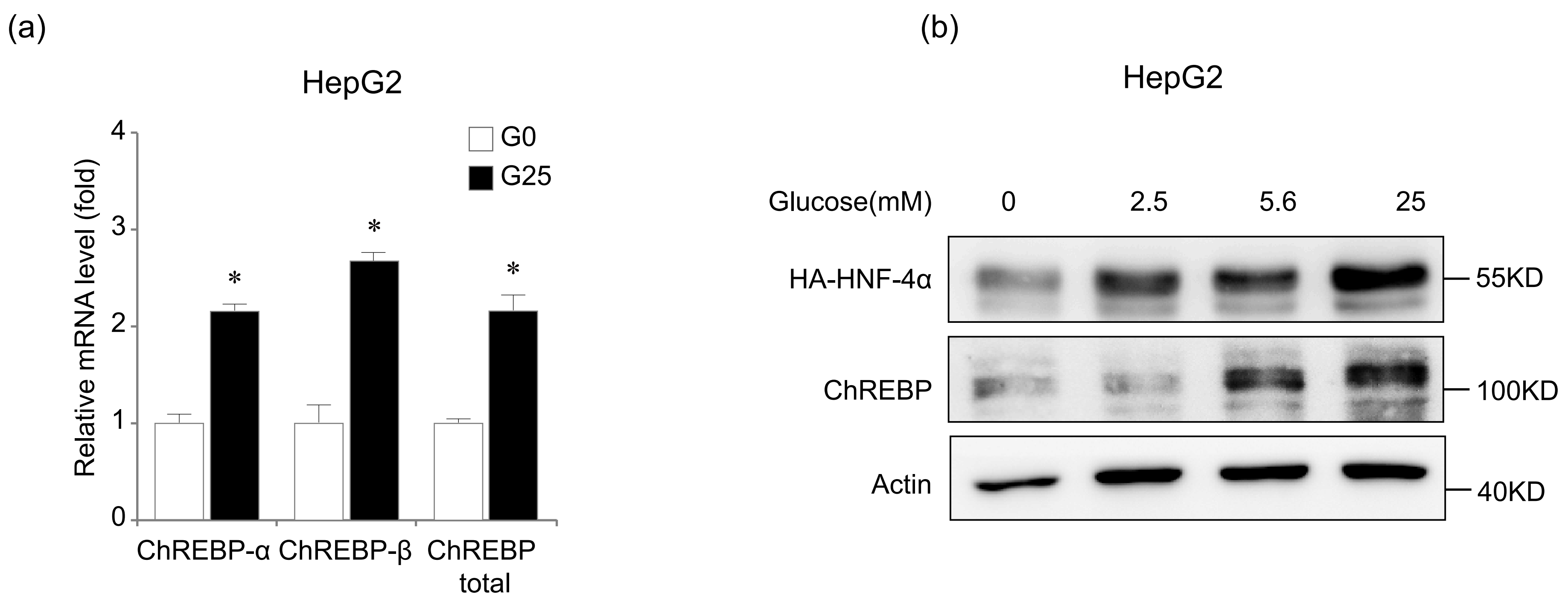

Supplementary table 1

(a)

Human	Forward (5'-3')	Reverse (5'-3')
β-Actin	GGACTTCGAGCAAGAGATGG	AGCACTGTGTTGGCGTACAG
ChREBP-α	AGTGCTTGAGCCTGGCCTAC	TTGTTCAGGCGGTCTTGC
ChREBP-β	AGCGGATTCCAGGTGAGG	TTGTTCAGGCGGATCTTGTC
ChREBP total	AACTGGAAGTTCTGGGTGTTC	AGGGAGTTCAGGACATTGG
ACC	TTGCTGCCTTACTTGGTGAT	GAGTGGTTTGGCATTGTGTC
LPK	TCAGCCCAGCTTCTGTCTC	CACGGAGCTTTCCACTTTC
SCD1	GTCCTTATGACAAGAACATTAGCC	AATCAATGAAGAATGTGGTGAAG
HNF-4α	ATCAGAAGGCACCAACC	TTGTCCACCACGCACT

(b)

Mouse	Forward (5'-3')	Reverse (5'-3')
18sRNA	TTGACTCAACACGGGAAACC	AGACAAATCGCCCACCAACACC
ChREBP-α	CGACACTCACCCACCTCTTC	TTGTTCAGCCGGATCTTGTC
ChREBP-β	TCTGCAGATCGCGTGGAG	CTTGTCCCGGCATAGCAAC
ChREBP total	ATCAGCGCTTTGACCAGATG	GGGAATTCAGGACAGTTGGC
FAS	GCTGCGGAAACTTCAGGAAAT	AGAGACGTGTCACTCCTGGACTT
LPK	TTGAGATCCCAGCAGAG AG	TGCATCTTTACAGCCTCCAC
SCD1	CCCTGCGGATCTTCCTTATC	TGTGTTTCTGAGAACTTGTGGTG
G6Pase	CTTGCTGCTCACTTTCCCC	TCCAAGCGCGAAACCAAAC
PEPCK	TGTGCACATCCAAACTCGC	TGAAGGCCTCGTACACCAG
HNF-4α	GATGCTTCTCGGAGGGTCTG	TGATGGCTGTGGAGTCTCG
